# Clinical efficacy of styloid incision truncation via percutaneous punching in treating styloid process syndrome

**DOI:** 10.1186/s13018-022-03486-7

**Published:** 2023-01-14

**Authors:** Yuebin Zheng, Bincheng Yan, Huacai Zhong, Wang Yi, Yirong Yang, Qian Wang

**Affiliations:** Department of Otorhinolaryngology, Head and Neck Surgery, Zigong First People’s Hospital, No. 42, Shangyi Haozhi Road, Zigong, 643000 Sichuan China

**Keywords:** Styloid process, Percutaneous punching, Styloid incision truncation, Clinical efficacy

## Abstract

**Objective:**

To clarify the clinical efficacy of styloid incision truncation via percutaneous punching in treating styloid process (styloid) syndrome.

**Methods:**

The clinical data of 40 styloid syndrome patients treated in our hospital from July 2018 to August 2021 were chosen and divided into an observation group and a control group in a random manner, with 20 cases in each. The control group received treatment with styloid truncation via an external cervical approach, and the observation group received treatment with styloid incision truncation via percutaneous punching. The operation time, intraoperative blood loss, length of truncated styloid, clinical efficacy, pain scores, postoperative complications and inflammatory cytokine levels were assessed in the both groups.

**Results:**

The intraoperative blood loss, operation time, length of truncated styloid and hospital stay in the observation group were significantly lower than those in the control group (*P* < 0.05). VAS pain scores were higher in both groups after the operation compared to before the operation. However, the observation group showed a statistically significant reduction in comparison with the control group (*P* < 0.05). The treatment effectiveness and complication rates of the two groups exhibited significant differences (*P* < 0.05). After the operation, TNF-α, CRP, and IL-6 levels in both groups were elevated compared to those before the operation. The observation group, however, showed significant depletion compared to the control group (*P* < 0.05).

**Conclusion:**

Styloid incision truncation via percutaneous punching was not only effective in treating styloid syndrome, but also caused less trauma and fewer complications. It promotes patient recovery and requires a simple operation, making it worthy of promotion in hospitals.

## Introduction

The styloid process (styloid) is a thin cylindrical bone located at the back and lower part of the petrous part of the temporal bone, anterior, and medial of the stylomastoid foramen, and running forward, downward, and inward [1]. A styloid length exceeding 2.5 cm means that the styloid is too long [2], and those with clinical symptoms are called styloid syndrome, also known as Eagle syndrome [3]. This disease is a general term for symptoms such as pharyngeal foreign body sensation, pharyngalgia or reflex earache, head and neck pain, saliva increase caused by long or abnormal styloid process or its location and shape stimulating adjacent vessels and nerves [4]. The cause of this condition is that the temporal bone styloid is abnormally long, oriented, and shaped, stimulating adjacent blood vessels and nerves, which can induce neurasthenia symptoms, such as tinnitus, salivation, insomnia, and others, and is most common in adult population [1, 5, 6]. Styloid truncation is an effective method for treating styloid syndrome [7]. Saliva increase caused by long or abnormal styloid process or its location and shape stimulating adjacent vessels and nerves the traditional transcatheter approach is to remove tonsils first and then expose the styloid. In recent years, our department has applied styloid incision truncation via percutaneous punching to treat styloid syndrome, receiving good results. Thus, we attempted to clarify the clinical efficacy of styloid incision truncation via percutaneous punching in treating styloid syndrome.

## Materials and methods

### General data

The clinical data of 40 patients with clinical symptoms relevant to styloid syndrome treated in our hospital from July 2018 to August 2021 were chosen and divided into a control group (styloid truncation via an external cervical approach) and an observation group (styloid incision truncation via percutaneous punching) in a random manner, with 20 cases in each group.

All cases underwent bilateral styloid CT and X-ray, measuring its length, orientation, and shape, confirming that the styloid is too long.

Inclusion criteria were as follows: (1) 18–65 years old, not limited to gender; (2) styloid syndrome diagnosed according to guideline criteria (symptoms were obviously on the abnormal side of styloid; styloid length measured by three-dimensional CT reconstruction was greater than 2.5 cm, and the styloid inclination angle was less than 70° or over 80°; (3) pharyngeal symptoms, unstable position, and obviously abnormal styloid, requiring surgery; (4) styloid length measured by styloid three-dimensional reconstruction was normal, but clinical symptoms were obvious, and patients had strong requirements for surgery.

Exclusion criteria were as follows: (1) Complicated with severe liver, kidney, cardiovascular and cerebrovascular abnormalities; (2) those with mental disorders and other diseases unable to cooperate with examination; (3) women who were breastfeeding, pregnant and menstrual; (4) had participated in any clinical trial in the past 3 months; (5) unable or unwilling to cooperate to complete follow-up.

### Treatment methods

The control group received treatment with styloid truncation via an external cervical approach. The patients were placed in the supine position with shoulder pads, the head tilted to the opposite side, and the jaw raised. Routine sterilization and draping were done after general anesthesia. Starting from the anterior and inferior aspects of the mastoid, at a transverse finger (1.5–2 cm) below the ascending branch of the mandible, a skin incision was made parallel to the anterior border of the sternocleidomastoid muscle to the plane of the hyoid. The tissue was bluntly dissected along the anterior border of the sternocleidomastoid muscle, and the digastric and stylohyoid muscles were examined. The tip and body of the styloid were probed upward along the stylohyoid muscle to expose its tip. Under direct vision, muscles and ligaments attached to the styloid were separated with a peeler and an ethmoid curette. The blood vessel was protected with a retractor, and the styloid was broken off with a needle holder near the root. A silicone drainage strip was placed in the surgical cavity, the incision was sutured layer by layer, and local pressure was applied for bandaging.

The observation group received treatment with styloid incision truncation via percutaneous punching. The patients were placed in the supine position with shoulder pads, the head tilted to the opposite side, and the jaw raised. Routine sterilization and draping were done after general anesthesia. On the neck, 2 cm from the mandibular border and the anterior border of the sternocleidomastoid muscle, the puncture head and sleeve were fastened. Percutaneous minimally invasive drilling was performed on the puncture slope, and an endoscope system placed on the sleeve was used to visualize and walk through the interstitial space to the styloid area. Blood vessels and nerves were avoided during the operation. After finding the styloid, the puncture head was withdrawn, and the sleeve was inserted into the sleeve from the distal end of the styloid. Moreover, the tissue attached to the styloid was peeled off, and the cutting device was put into the sleeve. The root of the styloid was cut off and removed from the discharge port. The device was withdrawn, and the skin incision was sutured.

### Observation indicators

The operation time, intraoperative blood loss and length of truncated styloid were compared between the two groups. The pain degree at 24 h after the operation was evaluated via the VAS method. The relief of local pain (pharyngalgia, cervicodynia, earache) and occurrence of complications in patients were observed.

Efficacy criteria: postoperative follow-up was performed 3–6 months, mean: 4.1 months. Cured: postoperative symptoms disappeared completely; improved: postoperative pharyngeal or other symptoms mostly disappeared, although sometimes occurred, but the degree was remarkably reduced; ineffective: postoperative symptoms did not improve or did not improve markedly. Effective rate = (cured + improved) cases/total cases × 100%.

Serum CRP, TNF-α, and IL-6 levels before and after the operation were compared between the groups. The detection methods of serum CRP, TNF-α, and IL-6 levels were as follows: 10 ml of fasting venous blood in the morning in both groups was collected before the operation and 3 days after the operation and centrifuged at 3000 r/min for 10 min. The levels of the above indicators were assessed via enzyme-linked immunosorbent assay (ELISA), and specific operations were carried out in accordance with the kit instructions. Relevant kits were provided by Shanghai Enzyme-linked Biotechnology Co., Ltd.

## Results

### General characteristics

In the control group, there were nine males and 11 females, aged 18–64 years old, mean: 43 years old; medical history was 5 months to 14 years. There were 15 cases of unilateral disease and five cases of bilateral disease. Clinical manifestations were as follows: nine cases of pharyngalgia, four cases of pharyngeal foreign body sensation, five cases of earache, one case of earache with headache, and one case of radiation cervicodynia; in unilateral disease, 11 cases had long styloid palpable by oropharyngeal examination, four cases had unpalpable styloid, and 1% lidocaine closure around tonsil fossa could temporarily relieve symptoms. In the observation group, there were ten males and ten females, aged 18–65 years old, mean: 44 years old; clinical symptoms of styloid syndrome was 6 months to 14 years. There were 16 cases of unilateral disease and four cases of bilateral disease. Clinical manifestations were as follows: ten cases of pharyngalgia, four cases of pharyngeal foreign body sensation, four cases of earache, two cases of earache with headache, and one case of radiation cervicodynia. In unilateral disease, 12 cases had palpable long styloid by oropharyngeal examination, four cases had unpalpable styloid, and 1% lidocaine closure around tonsil fossa could temporarily relieve symptoms.

All cases underwent bilateral styloid CT and X-ray, measuring its length, orientation, and shape, confirming that the styloid is too long. The left side was 3.0–5.9 cm, mean: 4.1 cm; the right side was 2.8–5.8 cm, mean: 3.9 cm. The longest styloid in the control group was 5.9 cm, and the shortest was 2.8 cm, while the longest styloid in the observation group was 5.8 cm, and the shortest was 2.9 cm. After statistical analysis, the general clinical data presented no difference between the two groups (*P* > 0.05).

### Comparison of surgical indicators between the two groups

**Fig. 1:**
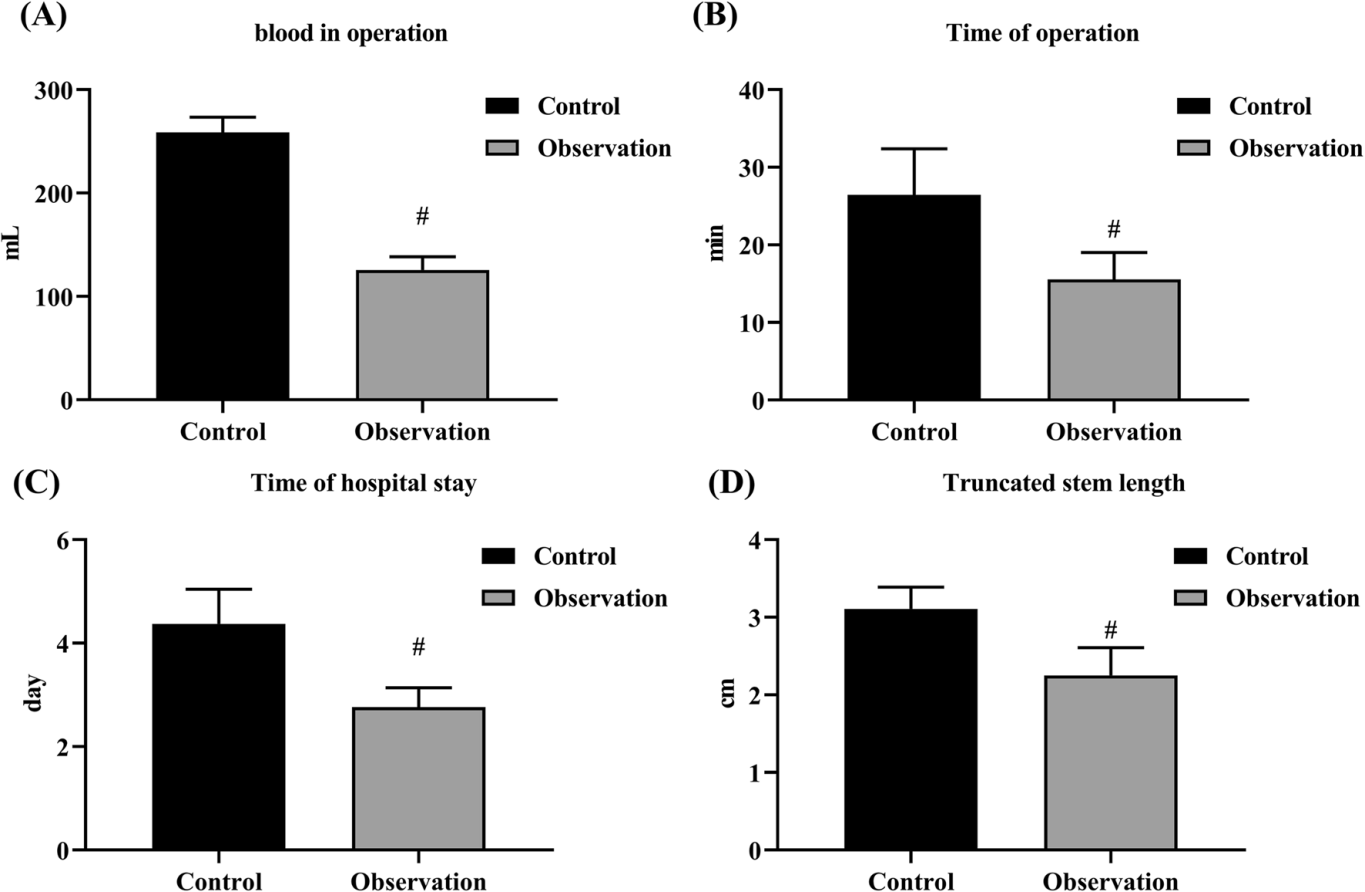
Surgical indicators in the control and observation groups. **A** Intraoperative blood loss. **B** Operation time. **C** Duration of hospital stay. **D** Length of truncated styloid. # indicates *P* < 0.05 versus control group

The intraoperative blood loss, operation time, length of truncated styloid and hospital stay in the observation group were significantly lower than those in the control group (*P* < 0.05) (Fig. 1A–D).

### Comparison of postoperative pain scores between the two groups

**Fig. 2:**
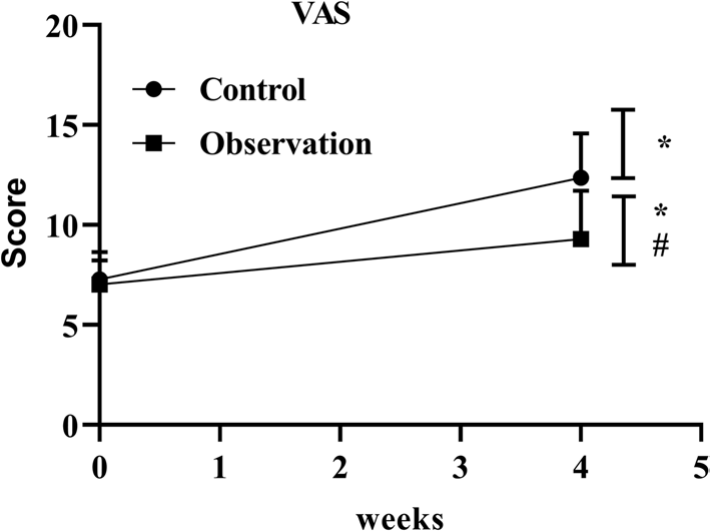
Postoperative VAS pain scores in the control and observation groups. #*P* < 0.05, versus control group; *indicates *P* < 0.05, versus before operation

Before the operation, VAS pain scores presented no difference between the two groups (*P* > 0.05). After the operation, VAS pain scores in both groups presented elevation relative to those before the operation, but the observation group presented depletion relative to the control group, with statistical significance (*P* < 0.05) (Fig. 2).

### Comparison of clinical efficacy between the two groups

**Fig. 3:**
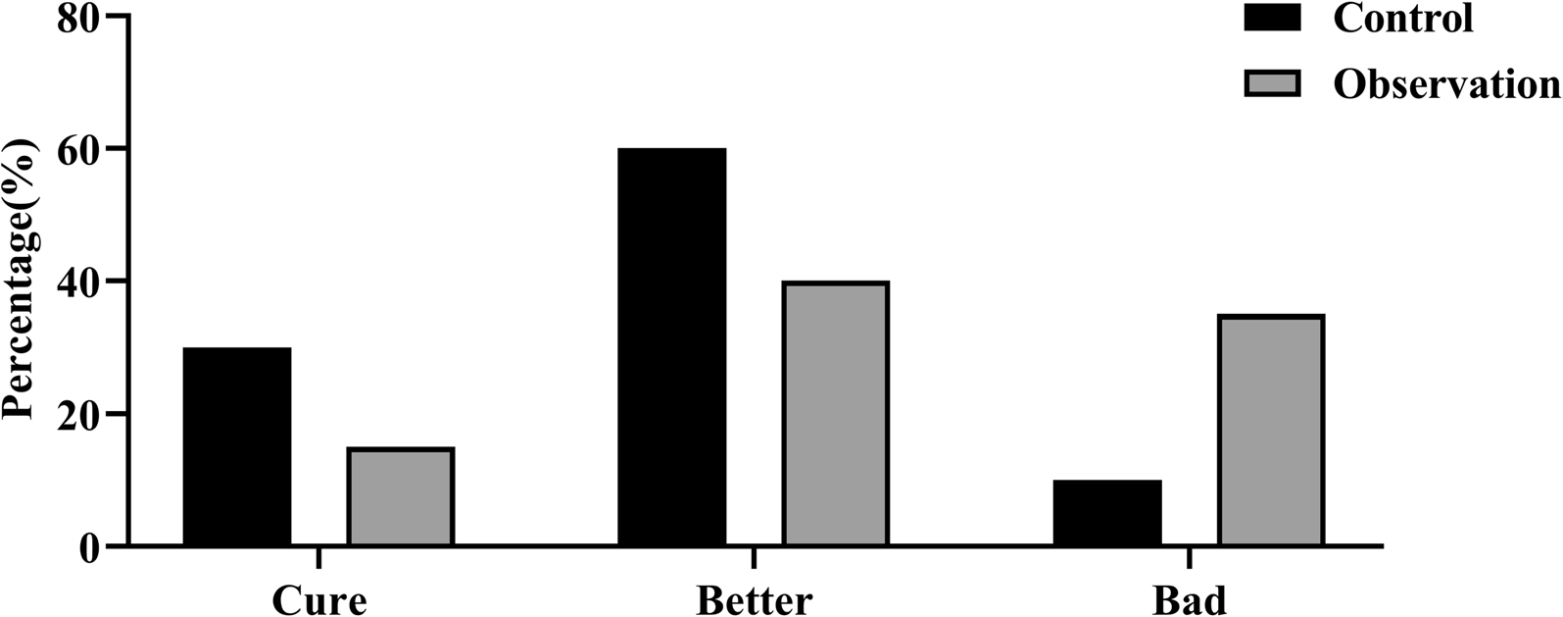
Clinical efficacy in the control and observation groups

After treatment, there were six cured and 12 improved patients in the observation group and three and eight in the control group, respectively. The treatment effective rates were significantly different between the two groups (*P* < 0.05) (Fig. 3).

### Comparison of postoperative complications between the two groups

There were six patients with local pain (pharyngalgia, cervicodynia, earache) in the observation group and 11 in the control group. The complication rates were significantly different between the two groups (*P* < 0.05) (Table 1).

**Table 1:** Postoperative complications in two groups

Groups	Pharyngalgia	Cervicodynia	Earache	Adverse reaction rate
Control group	2	3	1	30.00
Observation group	4	4	3	55.00
*t*	4.321			
*P*	< 0.001			

### Comparison of changes in serum CRP, TNF-α, and IL-6 levels between the two groups

**Fig. 4:**
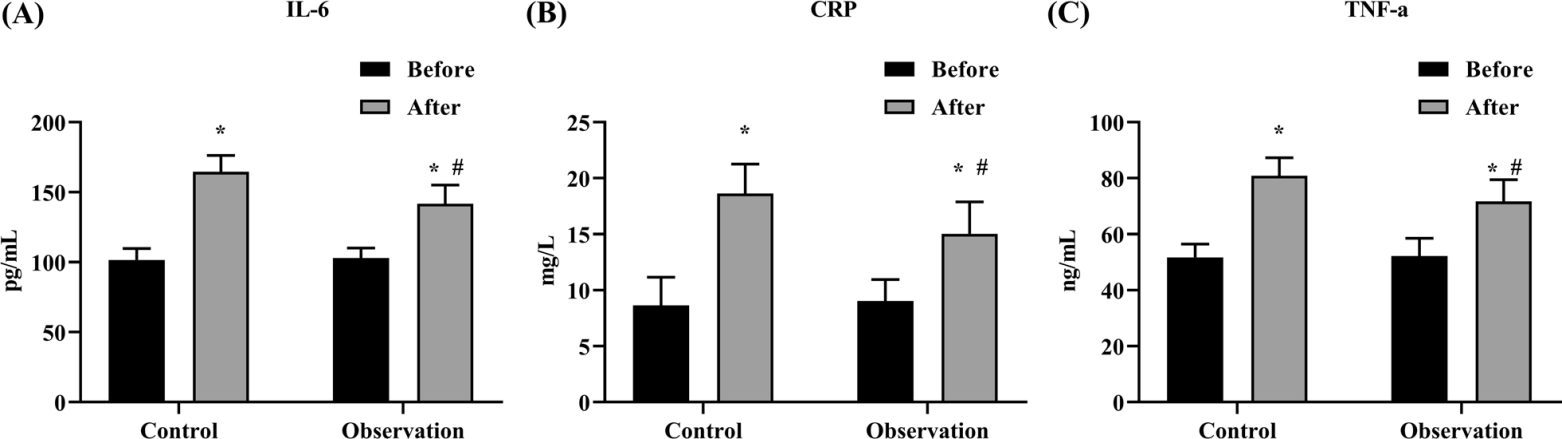
Comparison of changes in serum CRP, TNF-α, and IL-6 levels between control and observation groups. The levels of (**A**) IL-6, (**B**) CRP, and (**C**) TNF-α. # indicate *P* < 0.05, versus control group; *indicates *P* < 0.05, versus before operation

Before the operation, TNF-α, CRP, and IL-6 levels presented no difference between the two groups (*P* > 0.05). After the operation, TNF-α, CRP ,and IL-6 levels in both groups presented elevation relative to those before the operation, but the observation group presented depletion relative to the control group, with statistical significance (*P* < 0.05) (Fig. 4A–C).

## Discussion

The styloid is located at the junction of the bottom surface of the temporal bone's petrosal part and the mastoid process. It is slender and cylindrical, emerging from the front and inner sides of the stylomastoid foramen, and is attached to the stylopharyngeus, stylohyoid muscle, and stylohyoid ligaments as well as the stylomandibular ligament [8, 9]. Its root is similar to the facial nerve and stylomastoid artery emerging from the stylomastoid foramen, and the facial nerve runs on the posterolateral side of the styloid [10]. The middle and lower segments of the styloid are adjacent to the accessory nerve, hypoglossal nerve, vagus nerve, glossopharyngeal nerve, internal carotid artery, external carotid artery and their branches. The tip is just at the bifurcation of the superficial temporal and maxillary arteries from the external carotid artery. The styloid itself has no important physiological function. According to skull measurement data, its normal average length is 2.5 cm. The abnormal shape, orientation or length of the styloid can stimulate and compress surrounding blood vessels and nerves, causing pharyngalgia, cervicodynia, pharyngeal foreign body sensation, carotid artery compression symptoms, etc., which is called styloid process syndrome (styloid syndrome) [11]. Currently, styloid truncation is usually performed in clinical practice, generally including the transoropharyngeal tonsillar fossa approach and external cervical approach, each with its own advantages and disadvantages [7]. Among them, the external cervical approach can completely expose styloid and surrounding tissues, which is convenient for surgical operation, whereas wounds caused by such an approach are large, increasing the risk of nerve damage and infection as well as postoperative scar repair and other problems. Although the transoropharyngeal tonsillar fossa approach can overcome the above shortcomings, it must remove patients' tonsils and has the disadvantages of an insufficient intraoperative visual field and difficult identification and protection of surrounding tissues. A truncated styloid is not long enough to cause difficulties in eating in the future, greatly reducing its therapeutic efficacy, which is difficult to popularize on a large scale [12]. Thus, herein, we adopted a new surgical method of styloid incision truncation via percutaneous punching to treat styloid syndrome and clarified its therapeutic effect.

Medical research and clinical therapy revealed that the inflammatory response has a very direct relation to the functional recovery of patients' bodies [13, 14], and it is also a vital indicator affecting the recovery efficacy of patients' bodies after surgery, especially TNF-α, IL-6, and CRP, which are the main indicators in the actual treatment process. TNF-α is an inflammatory cytokine secreted by monocytes-macrophages [15]. When the body is stimulated by surgery, it elevates the activity of phagocytes in the local environment of the peritoneal cavity and induces the formation of various cytokines. From a physiological point of view, such a process will directly affect the defense function of the body. IL-6, a substance with a variety of biological activities, can better facilitate the secretion of inflammatory cells. Thus, the IL-6 level is also closely related to the degree of tissue damage. CRP is an acute phase response protein whose function is to directly reflect the degree of inflammation in the body [16, 17].

In the current study, 24 h after the operation, TNF-α, CRP, and IL-6 levels in both groups presented elevation relative to those before the operation, with statistical significance. TNF-α, CRP, and IL-6 levels in the observation group presented depletion relative to those in the control group, with statistical significance. The main reason was that the surgical wound in the observation group was relatively smaller, and its impact on the patient’s body was not great; thus, the inflammation was milder, which was beneficial to the patient’s subsequent recovery. Additionally, surgical wounds not only affect the occurrence of inflammation, but also cause postoperative pain and complications [18]. There were statistically significant differences between the two surgical procedures in terms of local pain, impact on speech, swallowing, operation time, and fever (complications) after the operation. Furthermore, the average length of styloid amputated by the external cervical approach was longer, the amount of bleeding was less through clinical observation, and hospital stays and hospitalization costs were also relatively low. This suggested that our styloid incision truncation via percutaneous punching can not only attenuate postoperative pain and complications of patients, but also reduce the difficulty of operation and save hospitalization costs of patients relative to traditional styloid truncation via an external cervical approach, which is conducive to hospital promotion.

In conclusion, styloid incision truncation via percutaneous punching in treating styloid syndrome not only has a good therapeutic effect, but also results in less trauma and fewer complications, conducive to the recovery of patients, with a simple operation, which is worthy of promotion in hospitals.

## Data Availability

The corresponding author can provide the data that support the findings of this study upon request.
